# Effect of the effluent released from the canine internal mammary artery after intraluminal and extraluminal perfusion of acetylcholine and adenosine diphosphate

**DOI:** 10.1186/1423-0127-16-45

**Published:** 2009-05-05

**Authors:** Nilce Mitiko Matsuda, Paul J Pearson, Hartzell V Schaff, Carlos E Piccinato, Alfredo J Rodrigues, Paulo Roberto Barbosa Evora

**Affiliations:** 1 Department of Surgery and Anatomy, Ribeirão Preto Faculty of Medicine, University of São Paulo, Ribeirão Preto, São Paulo, Brazil; 2 Division of Cardiovascular Surgery, Mayo Clinic Foundation, Rochester, Minnesota, USA

## Abstract

Segments of the canine internal mammary artery (35 mm in length) were suspended in vitro in an organ chamber containing physiological salt solution (95% O_2_/5% CO_2_, pH = 7.4, 37°C). Segments were individually cannulated and perfused at 5 ml/minute using a roller pump. Vasorelaxant activity of the effluent from the perfused internal mammary arteries was bioassayed by measuring the decrease in tension induced by the effluent of the coronary artery endothelium-free ring which had been contracted with prostaglandin F_2α _(2 × 10^-6 ^M). Intraluminal perfusion of adenosine diphosphate (10^-5 ^M) induced significant increase in relaxant activity in the effluent from the perfused blood vessel. However, when adenosine diphosphate (10^-5 ^M) was added extraluminally to the internal mammary artery, no change in relaxant activity in the effluent was noted. In contrast, acetylcholine produced significant increase in the relaxant activity on the effluent of the perfused internal mammary artery with both intraluminal and extraluminal perfusion. The intraluminal and extraluminal release of endothelium-derived relaxing factor (EDRF) by acetylcholine (10^-5 ^M) can be inhibited by site-specific administration of atropine (10^-5 ^M). These experiments indicate that certain agonists can induce the release of EDRF only by binding to intravascular receptors while other agonists can induce endothelium-dependent vasodilatation by acting on neural side receptors.

## Background

Accumulated evidence indicates that both the perivascular nerves located in the adventitia layer and endothelial cells control the tone of vascular smooth muscle [1,2]. Luminal release of the endothelium-derived relaxing factor (EDRF) or the endothelium-derived nitric oxide (EDNO) from the endothelium stimulated by acetylcholine has been extensively demonstrated [1,3-5].

Previous studies have been described that the coronary arteries are supplied by cholinergic nerves that modulate a non-adrenergic and non-cholinergic relaxation in isolated small coronary arteries suggesting that acetylcholine is also able to stimulate inhibitory non-adrenergic and non-cholinergic mediators released from the perivascular neural receptor [6-8].

Even though some authors have demonstrated that certain agonists induce the release of EDRF only by binding to endothelium side receptors [1,3-5], others have demonstrated inhibitory mediators released by agonists acting on perivascular nerves located in the adventitia layer [6-8]. Therefore, the purpose of our work was to determine the biologic activity of the effluent released from the canine internal mammary artery after intraluminal and extraluminal perfusion of acetylcholine and adenosine diphosphate. The biologic activity of the effluent released from the canine internal mammary artery was bioassayed on the coronary artery from which the endothelium had been previously removed and pre-contracted with prostaglandin.

## Materials and methods

### Tissue

According to the procedures and the handling of the animals approved by the Institutional Animal Care and Use Committee of the Mayo Foundation, mongrel dogs (25 to 30 Kg) of either sex were anesthetized with intravenously injected pentobarbital sodium (30 mg/kg bolus injection; Fort Dodge Laboratories, Fort Dodge, IA) and exsanguinated. The beating heart, internal mammary artery was excised and immersed in cold oxygenated physiologic salt solution with the following composition: NaCl, 118.3 mmol/L; KCl, 4.7 mmol/L; MgSO_4_, 1.2 mmol/L; KH_2_PO_4_, 1.22 mmol/L; CaCl_2_, 2.5 mmol/L; NaHCO_3_, 25.0 mmol/L; and glucose, 11.1 mmol/L.

### Bioassay experiments

The internal mammary artery was cleaned of connective tissue, with care taken not to touch the intimal surface. The biologic activity from the perfused of the internal mammary artery was bioassayed on the coronary artery ring from which the endothelium had been removed mechanically [9]. The internal mammary artery was perfused at a constant flow (5 ml/min), with the control solution (physiologic salt solution aerated with 95% O_2_/5% CO_2 _at 37°C). There was a transient delay of 1 second before the fluid reached the bioassay ring, which was suspended below the donor segment. The tension developed in the coronary bioassay ring was recorded with a force transducer (Grass FT03; Grass Instrument Company, Quincy, MA). The rings first were superfused for 60 minutes with control solution that passed through a stainless steel cannula (direct superfusion). During this time, the vessel was stretched progressively in a stepwise manner to its optimum length-tension relation (approximately 1.0 g). Control perfusion was provided from an aerated tower, and an adjacent aerated tower contained control solution plus prostaglandin F_2α _(2 × 10^-6 ^M).

Relaxations of the coronary artery endothelium-free ring were examined during contraction caused by prostaglandin F_2α _(2 × 10^-6 ^M). The experimental protocol was designed that the coronary artery endothelium-free ring was bathed with the solution released from the canine internal mammary artery. The time to record the relaxation was almost instantaneous. Once the relaxation reached its maximum, the coronary artery endothelium-free ring was perfused again with the effluent released from the canine internal mammary artery to recover to the prior level of the contraction. This procedure was repeated two, three times to confirm the relaxation (to rule out possible artifact). When the antagonists were added, the coronary artery endothelium-free ring was exposed to the compound for at least 15 minutes before changing the perfusion to the effluent released from the canine internal mammary artery.

### Drugs

The following drugs were used: acetylcholine chloride, atropine sulphate, pirenzepine, 4-(m-chlorophenylcarbamoyloxy)-2-butynyltrimethylammonium (McN-A-343), adenosine diphosphate, prostaglandin F_2α _(2 × 10^-6 ^M), obtained from the Sigma Chemical Company (St. Louis, MO), and N^G^-monomethyl-arginine (L-NMMA), obtained from Calbiochem Corp (La Jolla, CA). All drugs were prepared daily with distilled water. The concentrations were expressed as the final molar concentration in the organ chamber.

### Statistics

All data are expressed as mean ± SEM. In all experiments, n referred to the number of animals from which blood vessels were harvested. For bioassay experiments, relaxations were expressed as the percentage change in tension from the contraction of the bioassay ring in response to prostaglandin F_2α _(2 × 10^-6 ^M). Statistical evaluation of the data was performed by analysis of variance and Student's t test for either paired or unpaired observations. Values were considered statistically significant when p < 0.05. Relaxations were expressed as a percentage of reduction of the steady state tension developed after equilibration period tension of individual preparations.

## Results

**Figure 1:**
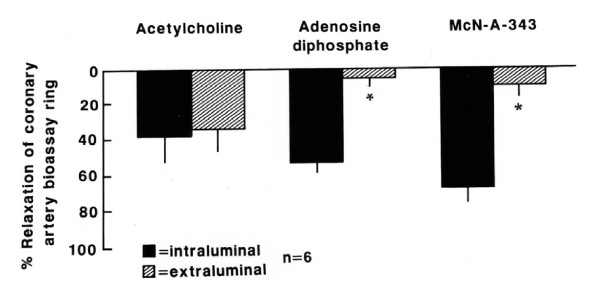
**Effect of the effluent from canine internal mammary artery on coronary artery endothelium-free ring**. Relaxation of the coronary artery endothelium-free ring induced by the effluent released from canine internal mammary artery stimulated by intraluminal and extraluminal perfusion of acetylcholine (10^-5 ^M), intraluminal and extraluminal adenosine diphosphate (10^-5 ^M) and intraluminal and extraluminal McN-A-343 (10^-5 ^M). Values represent mean ± SEM; n = 6. Relaxation magnitude is expressed as % of initial tonus. * p < 0.05.

The effluent released from the canine internal mammary artery produced relaxation of the coronary artery endothelium-free ring pre-contracted with prostaglandin when stimulated by both intraluminal and extraluminal perfusion of acetylcholine (10^-5 ^M) but only by intraluminal perfusion of adenosine diphosphate (10^-5 ^M) and McN-A-343 (10^-5 ^M, Figure 1, n = 6; p < 0.05).

**Figure 2:**
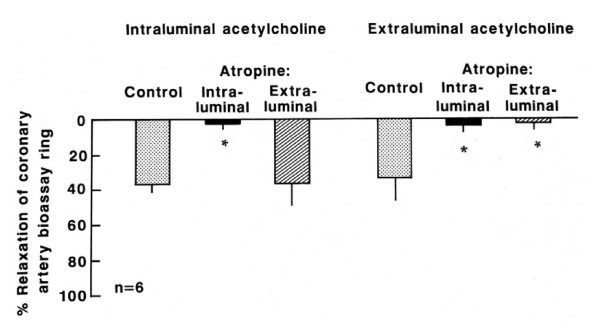
**Effect of the effluent from canine internal mammary artery on coronary artery endothelium-free ring**. Relaxation of the coronary artery endothelium-free ring induced by the effluent released from canine internal mammary artery stimulated by intraluminal and extraluminal perfusion of acetylcholine (10^-5 ^M) before (control) and after intraluminal and extraluminal atropine (10^-5 ^M). Values represent mean ± SEM; n = 6. Relaxation magnitude is expressed as % of initial tonus. * p < 0.05.

**Figure 3:**
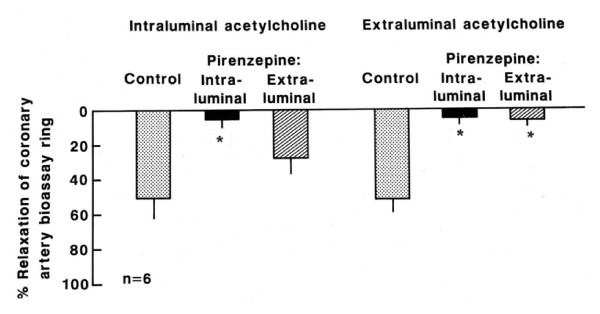
**Effect of the effluent from canine internal mammary artery on coronary artery endothelium-free ring**. Relaxation of the coronary artery endothelium-free ring induced by the effluent released from canine internal mammary artery stimulated by intraluminal and extraluminal perfusion of acetylcholine (10^-5 ^M) before (control) and after intraluminal and extraluminal pirenzepine (10^-5 ^M). Values represent mean ± SEM; n = 6. Relaxation magnitude is expressed as % of initial tonus. * p < 0.05.

**Figure 4:**
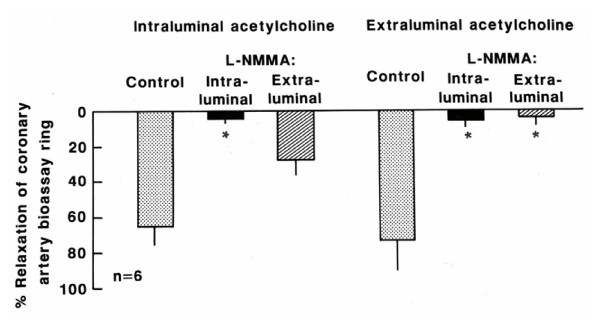
**Effect of the effluent from canine internal mammary artery on coronary artery endothelium-free ring**. Relaxation of the coronary artery endothelium-free ring induced by the effluent released from canine internal mammary artery stimulated by intraluminal and extraluminal perfusion of acetylcholine (10^-5 ^M) before (control) and after intraluminal and extraluminal L-NMMA (10^-4 ^M). Values represent mean ± SEM; n = 6. Relaxation magnitude is expressed as % of initial tonus. * p < 0.05.

The relaxation of the coronary artery endothelium-free ring pre-contracted with prostaglandin caused by the effluent released from the canine internal mammary artery stimulated by intraluminal perfusion of acetylcholine (10^-5 ^M) was inhibited only by intraluminal treatment with atropine (10^-5 ^M, Figure 2, n = 6; p < 0.05), pirenzepine (10^-5 ^M, Figure 3, n = 6; p < 0.05) and L-NNA (10^-4 ^M, Figure 4, n = 6; p < 0.05). On the other hand, the relaxation stimulated by extraluminal perfusion of acetylcholine (10^-5 ^M) was inhibited by both the intraluminal and extraluminal treatment with atropine (10^-5 ^M, Figure 2, n = 6; p < 0.05), pirenzepine (10^-5 ^M, Figure 3, n = 6; p < 0.05) and L-NNA (10^-4 ^M, Figure 4, n = 6; p < 0.05).

## Discussion

Vascular smooth muscle tissue is surrounded internally by the endothelium cells layer and externally by the adventitia layer containing sympathetic, parasympathetic and sensorial nerves [1,2,10]. It has been proposed also that NO is a messenger mediating vascular smooth muscle relaxation released by the activation of endothelium by acetylcholine and NANC mediators released from nerves [3-8]. Our results also suggest that acetylcholine increased both nerve- and endothelium-dependent EDNO release since acetylcholine produced significant increase in the relaxant activity of the effluent of the perfused internal mammary artery with both intraluminal and extraluminal perfusion and a NO-synthase inhibitor L-NMMA inhibited both.

Even though it has been demonstrated that both NO and ATP can be released from NANC nerves [11], the inhibitory response in circular smooth muscle of chicken anterior mesenteric artery was caused only by NO released from endothelium cells stimulated by neuronally ATP and not by NO or ATP released directly from perivascular NANC nerves [12]. In our experiments, intraluminal perfusion of adenosine diphosphate induced significant increase in relaxant activity in the effluent from the perfused blood vessel while extraluminal perfusion of adenosine diphosphate caused no change in relaxant activity of the effluent, suggesting that purinergic receptor related to EDNO release is present only on endothelium cells of canine internal mammary artery.

It has been demonstrated that muscarinic receptors mediate diverse effects on the vasculature and three major subtypes of receptors are present in endothelium cells, nervous tissue and also smooth muscle cells [13-15]. While M1 receptors contract canine venous smooth muscle tissue, M3 receptors contract porcine and bovine coronary arteries and rabbit aorta smooth muscle [13,14]. And also both M1 and M3 receptors mediate EDRF-dependent relaxant responses in canine coronary artery and rabbit aorta respectively [14,15]. In our experiments, acetylcholine seemed to act on the endothelium cells and nerves of the canine internal mammary artery by different muscarinic receptor since atropine inhibited EDRF release by both intraluminal and extraluminal perfusion of acetylcholine whereas McN-A-343 stimulated EDRF release only by intraluminal perfusion.

Intraluminal release of EDRF was stimulated by acetylcholine, McN-A-343 and adenosine diphosphate while extraluminal release of EDRF was stimulated only by acetylcholine. And both intraluminal and extraluminal perfusion of acetylcholine were inhibited by intraluminal but not by extraluminal perfusion of a NO-synthase inhibitor L-NMMA, suggesting that extraluminal perfusion of acetylcholine stimulated muscarinic receptor on nerves while intraluminal perfusion of acetylcholine stimulated muscarinic receptor on endothelium cells and both adventitia layer and endothelial cells activation stimulated EDNO release only from endothelium.

These experiments indicate that certain agonists can induce EDRF release from canine internal mammary artery only by binding on the endothelium surface receptors (direct effect), while other agonists can induce EDRF-dependent vasodilatation by acting on the adventitia surface receptors (indirect effect).

## Competing interests

The authors declare that they have no competing interests.

## Authors' contributions

NMM has been involved in analysis and interpretation of data, drafting the manuscript and acquisition of funding to prepare the manuscript. PJP helped to design the study and collecting data. HVS helped to design the study and collecting data. CEP helped to design the study. AJR helped to design the study. PRBE participated in the design of the study, collecting data and revising the manuscript and has given final approval of the version to be published.
